# Cardiac risk stratification and adverse outcomes in surgically managed patients with isolated traumatic spine injuries

**DOI:** 10.1007/s00068-023-02413-7

**Published:** 2024-01-03

**Authors:** Ahmad Mohammad Ismail, Maximilian Peter Forssten, Frank Hildebrand, Babak Sarani, Ioannis Ioannidis, Yang Cao, Marcelo A. F. Ribeiro, Shahin Mohseni

**Affiliations:** 1 School of Medical Sciences, Orebro University, 701 82 Orebro, Sweden; 2 Department of Orthopedic Surgery, Orebro University Hospital, 701 85 Orebro, Sweden; 3 Department of Orthopedics, Trauma and Reconstructive Surgery, University Hospital RWTH Aachen, Pauwelsstrasse 30, 52074 Aachen, Germany; 4 Center of Trauma and Critical Care, George Washington University, Washington, DC USA; 5 Clinical Epidemiology and Biostatistics, School of Medical Sciences, Faculty of Medicine and Health, Orebro University, 701 82 Orebro, Sweden; 6 Division of Trauma, Critical Care & Acute Care Surgery, Department of Surgery, Sheikh Shakhbout Medical City, Mayo Clinic, Abu Dhabi, United Arab Emirates; 7 Pontifical Catholic University of São Paulo, São Paulo, Brazil; 8 Khalifa University and Gulf Medical University, Abu Dhabi, United Arab Emirates

**Keywords:** Traumatic spine injury, Revised Cardiac Risk Index, Mortality, Cardiopulmonary complications, Risk stratification

## Abstract

**Introduction:**

As the incidence of traumatic spine injuries has been steadily increasing, especially in the elderly, the ability to categorize patients based on their underlying risk for the adverse outcomes could be of great value in clinical decision making. This study aimed to investigate the association between the Revised Cardiac Risk Index (RCRI) and adverse outcomes in patients who have undergone surgery for traumatic spine injuries.

**Methods:**

All adult patients (18 years or older) in the 2013–2019 TQIP database with isolated spine injuries resulting from blunt force trauma, who underwent spinal surgery, were eligible for inclusion in the study. The association between the RCRI and in-hospital mortality, cardiopulmonary complications, and failure-to-rescue (FTR) was determined using Poisson regression models with robust standard errors to adjust for potential confounding.

**Results:**

A total of 39,391 patients were included for further analysis. In the regression model, an RCRI ≥ 3 was associated with a threefold risk of in-hospital mortality [adjusted IRR (95% CI): 3.19 (2.30–4.43), *p* < 0.001] and cardiopulmonary complications [adjusted IRR (95% CI): 3.27 (2.46–4.34), *p* < 0.001], as well as a fourfold risk of FTR [adjusted IRR (95% CI): 4.27 (2.59–7.02), *p* < 0.001], compared to RCRI 0. The risk of all adverse outcomes increased stepwise along with each RCRI score.

**Conclusion:**

The RCRI may be a useful tool for identifying patients with traumatic spine injuries who are at an increased risk of in-hospital mortality, cardiopulmonary complications, and failure-to-rescue after surgery.

## Introduction

Traumatic spine injuries are a significant cause of permanent disability and mortality, with the United States having among the highest incidence rate of spinal trauma in the world [1–6]. Spinal injuries, especially those affecting the spinal cord have a particularly high mortality rate, with up to 16% of patients dying within 30 days of injury and 32% within 1 year of admission [2, 7, 8]. While surgical treatment has been associated with lower mortality compared to non-operative management [8, 9], patients with operatively managed spinal trauma remain at high risk of postoperative morbidity and mortality [10]. Particularly among older adults, the incidence of traumatic spine injuries has been steadily increasing [6, 11, 12]. This is especially concerning given that older patients are at a disproportionate risk of mortality following injury, likely due to a higher incidence of frailty and comorbidity among this population [4, 5]. Given that the primary causes of mortality after spinal injury are of cardiopulmonary origin [13, 14], the ability to categorize patients based on their underlying risk for the adverse outcomes could be of great value in clinical decision making.

The Revised Cardiac Risk Index (RCRI) is a risk stratification tool used to predict adverse outcomes within 30 days after noncardiac surgery [15, 16]. It has been validated in various populations where higher RCRI scores have been associated with increased mortality and other adverse outcomes, including patients with severe traumatic brain injury [17]. rib fractures [18], and those with traumatic hip fractures [19, 20]. among others [21]. Although the RCRI has not been specifically assessed as a stratification tool for patients with isolated traumatic spine injuries, it may be able to accurately reflect the effect of multimorbidity in this population. In this study, the aim was to determine whether the RCRI could be used to risk-stratify surgically managed patients who have suffered a traumatic spine injury without concomitant injuries, with the hypothesis that an elevated cardiac risk, measured by the RCRI, would be associated with an increased risk of in-hospital mortality, cardiopulmonary complications, and failure-to-rescue.

## Methods

Due to the utilization of an anonymized, retrospective dataset for all analyses, ethical approval from an institutional review board was not required for the current study. All aspects of the investigation were performed in compliance with the Declaration of Helsinki and the Strengthening the Reporting of Observational Studies in Epidemiology (STROBE) guidelines [22]. Data were collected from the American College of Surgeons Trauma Quality Improvement Program (TQIP) database and included demographic information, injury details, surgical procedures, discharge disposition, and complications [23]. Only adult patients (18 years or older) with isolated spine injuries resulting from blunt force trauma, who underwent spinal surgery, were eligible for inclusion in the study. An isolated spine injury was defined as any spine Abbreviated Injury Score (AIS) > 1 with an AIS ≤ 1 in all other body regions. Exclusion criteria included surgery occurring more than five days after admission and a Spine AIS of 6, which would generally not be considered survivable.

### Calculating the Revised Cardiac Risk Index

The Revised Cardiac Risk Index (RCRI) is a tool traditionally used to predict the 30-day risk of postoperative myocardial infarction, cardiac arrest, and all-cause mortality [15, 16]. The RCRI was determined using six dichotomous variables: previous ischemic heart disease, previous cerebrovascular disease, congestive heart failure, renal insufficiency (defined as chronic kidney disease or acute kidney injury), and high-risk surgery [17–20]. High-risk surgery is defined as any suprainguinal vascular surgery as well as intrathoracic and intraperitoneal surgery, according to the American College of Cardiology and American Heart Association [24]. Although spine surgery may be considered a high risk surgery, patients were not awarded a point due to it not being included in the generally accepted definition; however, since all patients in the studied cohort underwent spine surgery this decision does not change the overall results.

### Statistical analysis

The study population was divided into four groups based on their RCRI scores (0, 1, 2, or ≥ 3) [18]. Continuous variables were summarized using median values and interquartile ranges (IQRs), while categorical variables were presented as counts and percentages. The Kruskal–Wallis test was employed to evaluate the statistical significance of differences between continuous variables, and the Chi-squared test or Fisher's exact test was utilized for categorical variables, as appropriate. The primary outcome of interest was in-hospital mortality, while secondary outcomes included in-hospital cardiopulmonary complications (myocardial infarction, cardiac arrest with CPR, stroke, pulmonary embolism, acute respiratory distress syndrome, or pneumonia), and failure-to-rescue (FTR), defined as in-hospital mortality following a cardiopulmonary complication.

To investigate the association between cardiac risk and the previously listed adverse outcomes while controlling for potential confounding variables, Poisson regression models with robust standard errors were employed. In these models, in-hospital mortality, complications, or FTR served as the response variables, while the explanatory variables consisted of cardiac risk (measured using the RCRI), as well as age, sex, race, highest AIS in each region, level of injury, presence and degree of spinal cord injury, level of spine surgery, comorbidities (hypertension, history of peripheral vascular disease, dementia, currently receiving chemotherapy for cancer, metastatic cancer, chronic obstructive pulmonary disease, smoking status, cirrhosis, coagulopathy, drug use disorder, alcohol use disorder, major psychiatric illness), and advanced directives limiting care. The results are presented as adjusted incidence rate ratios (IRRs) with corresponding 95% confidence intervals (CIs).

A two-sided p-value of less than 0.05 was considered statistically significant. Among the patients in the current dataset, less than 2% had missing data, and were assumed to be missing at random. To handle missing data, multiple imputation by chained equations was used, with logistic regression for sex and race and a proportional odds model for the spine AIS. The statistical software R 4.0.5 (R Foundation for Statistical Computing, Vienna, Austria) was used to perform the analyses, with the packages *tidyverse*, *haven*, *mice*, and *robustbase* [25].

## Results

After applying the previously mentioned inclusion and exclusion criteria, a total of 39,391 patients were eligible for further analysis. Patients with an elevated cardiac risk were generally older (RCRI ≥ 3 vs RCRI 0: 72 vs 50 years, *p* < 0.001), less likely to be female (RCRI ≥ 3 vs RCRI 0: 25.3% vs 32.1%, *p* = 0.013), more likely to have advanced directives limiting care (RCRI ≥ 3 vs RCRI 0: 5.3% vs 1.4%, *p* < 0.001), and had a higher prevalence of most comorbidities (Table 1). Patients with an elevated cardiac risk also tended to have more severe injuries on average (RCRI ≥ 3 vs RCRI 0, Spine AIS ≥ 4: 39.2% vs 24.2%, *p* < 0.001). Patients with an elevated cardiac risk were more likely to have suffered a cervical spine injury (RCRI ≥ 3 vs RCRI 0: 68.2%% versus 60.0%, *p* < 0.001) and a cervical spinal cord injury (42.0% versus 29.6%, *p* < 0.001) (Table 2).

**Table 1:** Demographics of patients with surgically managed isolated traumatic spine injuries

	RCRI 0 (*N* = 30,581)	RCRI 1 (*N* = 7254)	RCRI 2 (*N* = 1311)	RCRI ≥ 3 (*N* = 245)	*P*-value
Age, median [IQR]	50 [33–64]	66 [56–76]	71 [62–78]	72 [64–78]	< 0.001
Sex, *n* (%)					0.013
Female	9810 (32.1)	2237 (30.8)	394 (30.1)	62 (25.3)	
Male	20,763 (67.9)	5,016 (69.1)	917 (69.9)	183 (74.7)	
Missing	8 (0.0)	1 (0.0)	0 (0.0)	0 (0.0)	
Race, n (%)					
White	23,841 (78.0)	5,591 (77.1)	1,010 (77.0)	194 (79.2)	0.183
Black	3,373 (11.0)	910 (12.5)	190 (14.5)	36 (14.7)	< 0.001
Asian	649 (2.1)	165 (2.3)	30 (2.3)	4 (1.6)	0.790
American Indian	261 (0.9)	61 (0.8)	8 (0.6)	0 (0.0)	0.493
Pacific Islander	79 (0.3)	24 (0.3)	6 (0.5)	2 (0.8)	0.088
Other	1853 (6.1)	382 (5.3)	52 (4.0)	9 (3.7)	< 0.001
Missing	346 (1.1)	65 (0.9)	10 (0.8)	0 (0.0)	
Hypertension, *n* (%)	8228 (26.9)	4978 (68.6)	1081 (82.5)	199 (81.2)	< 0.001
History of angina, *n* (%)	0 (0.0)	20 (0.3)	10 (0.8)	5 (2.0)	< 0.001
Previous myocardial infarction, *n* (%)	0 (0.0)	138 (1.9)	113 (8.6)	75 (30.6)	< 0.001
Congestive heart failure, *n* (%)	0 (0.0)	527 (7.3)	528 (40.3)	167 (68.2)	< 0.001
History of peripheral vascular disease, *n* (%)	83 (0.3)	87 (1.2)	48 (3.7)	15 (6.1)	< 0.001
Cerebrovascular disease, *n* (%)	0 (0.0)	363 (5.0)	271 (20.7)	88 (35.9)	< 0.001
Dementia, *n* (%)	524 (1.7)	294 (4.1)	91 (6.9)	21 (8.6)	< 0.001
Currently receiving chemotherapy for cancer, *n* (%)	69 (0.2)	31 (0.4)	5 (0.4)	0 (0.0)	0.013
Metastatic cancer, *n* (%)	148 (0.5)	49 (0.7)	13 (1.0)	4 (1.6)	0.001
COPD, *n* (%)	1412 (4.6)	691 (9.5)	248 (18.9)	58 (23.7)	< 0.001
Current smoker, *n* (%)	8017 (26.2)	1250 (17.2)	172 (13.1)	23 (9.4)	< 0.001
Chronic renal failure, *n* (%)	0 (0.0)	165 (2.3)	205 (15.6)	97 (39.6)	< 0.001
Acute kidney injury, *n* (%)	0 (0.0)	91 (1.3)	78 (5.9)	34 (13.9)	< 0.001
Diabetes mellitus, *n* (%)	0 (0.0)	5093 (70.2)	1108 (84.5)	230 (93.9)	< 0.001
Cirrhosis, *n* (%)	197 (0.6)	92 (1.3)	25 (1.9)	6 (2.4)	< 0.001
Coagulopathy, *n* (%)	618 (2.0)	409 (5.6)	121 (9.2)	26 (10.6)	< 0.001
Drug use disorder, *n* (%)	2129 (7.0)	308 (4.2)	41 (3.1)	7 (2.9)	< 0.001
Alcohol use disorder, *n* (%)	2620 (8.6)	529 (7.3)	73 (5.6)	6 (2.4)	< 0.001
Major psychiatric illness, *n* (%)	3023 (9.9)	906 (12.5)	189 (14.4)	43 (17.6)	< 0.001
Advanced directive limiting care, *n* (%)	428 (1.4)	296 (4.1)	85 (6.5)	13 (5.3)	< 0.001

*COPD* chronic obstructive pulmonary disease

**Table 2:** Clinical characteristics of patients with surgically managed isolated traumatic spine injuries

	RCRI 0 (*N* = 30,581)	RCRI 1 (*N* = 7254)	RCRI 2 (*N* = 1311)	RCRI ≥ 3 (*N* = 245)	*P*-value
Head AIS, *n* (%)					0.034
Injury not present	26,399 (86.3)	6201 (85.5)	1103 (84.1)	206 (84.1)	
1	4182 (13.7)	1053 (14.5)	208 (15.9)	39 (15.9)	
Face AIS, *n* (%)					< 0.001
Injury not present	25,454 (83.2)	5895 (81.3)	1055 (80.5)	188 (76.7)	
1	5,127 (16.8)	1,359 (18.7)	256 (19.5)	57 (23.3)	
Neck AIS, *n* (%)					0.084
Injury not present	30,176 (98.7)	7151 (98.6)	1283 (97.9)	243 (99.2)	
1	405 (1.3)	103 (1.4)	28 (2.1)	2 (0.8)	
Spine AIS, *n* (%)					< 0.001
2	11,515 (37.7)	2,555 (35.2)	480 (36.6)	83 (33.9)	
3	11,647 (38.1)	2444 (33.7)	429 (32.7)	66 (26.9)	
4	5452 (17.8)	1,590 (21.9)	273 (20.8)	60 (24.5)	
5	1952 (6.4)	664 (9.2)	129 (9.8)	36 (14.7)	
Missing	15 (0.0)	1 (0.0)	0 (0.0)	0 (0.0)	
Thorax AIS, *n* (%)					0.411
Injury not present	28,683 (93.8)	6839 (94.3)	1227 (93.6)	228 (93.1)	
1	1898 (6.2)	415 (5.7)	84 (6.4)	17 (6.9)	
Abdomen AIS, *n* (%)					0.121
Injury not present	29,692 (97.1)	7067 (97.4)	1284 (97.9)	236 (96.3)	
1	889 (2.9)	187 (2.6)	27 (2.1)	9 (3.7)	
Upper extremity AIS, *n* (%)					0.026
Injury not present	27,304 (89.3)	6523 (89.9)	1143 (87.2)	221 (90.2)	
1	3,277 (10.7)	731 (10.1)	168 (12.8)	24 (9.8)	
Lower extremity AIS, *n* (%)					0.262
Injury not present	27,565 (90.1)	6593 (90.9)	1188 (90.6)	220 (89.8)	
1	3,016 (9.9)	661 (9.1)	123 (9.4)	25 (10.2)	
External/Other AIS, *n* (%)					0.038
Injury not present	29,258 (95.7)	6,957 (95.9)	1,271 (96.9)	240 (98.0)	
1	1,323 (4.3)	297 (4.1)	40 (3.1)	5 (2.0)	
Level of spine injury, *n* (%)					
Cervical	18,348 (60.0)	4680 (64.5)	879 (67.0)	167 (68.2)	< 0.001
Thoracic	8345 (27.3)	2,067 (28.5)	399 (30.4)	84 (34.3)	0.002
Lumbar	8918 (29.2)	1600 (22.1)	245 (18.7)	34 (13.9)	< 0.001
Cervical spinal cord injury, *n* (%)	9066 (29.6)	2746 (37.9)	512 (39.1)	103 (42.0)	< 0.001
Transient neurological signs	1508 (4.9)	384 (5.3)	74 (5.6)	9 (3.7)	0.302
Incomplete spinal cord injury	5117 (16.7)	1,546 (21.3)	265 (20.2)	57 (23.3)	< 0.001
Complete spinal cord injury	1177 (3.8)	462 (6.4)	88 (6.7)	22 (9.0)	< 0.001
Unknown degree	1264 (4.1)	354 (4.9)	85 (6.5)	15 (6.1)	< 0.001
Thoracic spinal cord injury, *n* (%)	1759 (5.8)	421 (5.8)	72 (5.5)	27 (11.0)	0.006
Transient neurological signs	419 (1.4)	102 (1.4)	18 (1.4)	5 (2.0)	0.765
Incomplete spinal cord injury	562 (1.8)	123 (1.7)	19 (1.4)	7 (2.9)	0.342
Complete spinal cord injury	361 (1.2)	88 (1.2)	14 (1.1)	6 (2.4)	0.329
Unknown degree	417 (1.4)	108 (1.5)	21 (1.6)	9 (3.7)	0.028
Lumbar spinal cord injury, *n* (%)	1630 (5.3)	231 (3.2)	26 (2.0)	6 (2.4)	< 0.001
Transient neurological signs	715 (2.3)	97 (1.3)	9 (0.7)	4 (1.6)	< 0.001
Incomplete spinal cord injury	333 (1.1)	49 (0.7)	6 (0.5)	0 (0.0)	< 0.001
Complete spinal cord injury	117 (0.4)	17 (0.2)	3 (0.2)	1 (0.4)	0.186
Unknown degree	465 (1.5)	68 (0.9)	8 (0.6)	1 (0.4)	< 0.001
Level of spine surgery, *n* (%)					
Cervical	22,123 (72.3)	5315 (73.3)	967 (73.8)	180 (73.5)	0.306
Thoracic	16,898 (55.3)	3789 (52.2)	680 (51.9)	114 (46.5)	< 0.001
Lumbar	14,596 (47.7)	2859 (39.4)	469 (35.8)	65 (26.5)	< 0.001

*AIS* abbreviated injury severity score

Patients with an elevated cardiac risk demonstrated higher crude rates of in-hospital mortality (RCRI ≥ 3 vs RCRI 0: 14.7% vs 1.2%, *p* < 0.001), cardiopulmonary complications (RCRI ≥ 3 vs RCRI 0: 18.0% vs 3.1%, *p* < 0.001), and FTR (RCRI ≥ 3 vs RCRI 0: 7.3% vs 0.5%, *p* < 0.001). All of the specific complications recorded were also more prevalent among patients with an elevated cardiac risk (Table 3). After adjusting for confounding, an RCRI ≥ 3 was associated with a threefold risk of in-hospital mortality [adjusted IRR (95% CI): 3.19 (2.30–4.43), *p* < 0.001] and cardiopulmonary complications [adjusted IRR (95% CI): 3.27 (2.46–4.34), *p* < 0.001], as well as a fourfold risk of FTR [adjusted IRR (95% CI): 4.27 (2.59–7.02), *p* < 0.001], compared to RCRI 0. The risk of all adverse outcomes increased stepwise along with the RCRI (Table 4).

**Table 3:** Crude outcomes in patients with surgically managed isolated traumatic spine injuries

	RCRI 0 (*N* = 30,581)	RCRI 1 (*N* = 7254)	RCRI 2 (*N* = 1311)	RCRI ≥ 3 (*N* = 245)	*P*-value
In-hospital mortality, *n* (%)	374 (1.2)	282 (3.9)	107 (8.2)	36 (14.7)	< 0.001
Cardiopulmonary complication, *n* (%)	942 (3.1)	497 (6.9)	167 (12.7)	44 (18.0)	< 0.001
Myocardial infarction, *n* (%)	35 (0.1)	36 (0.5)	18 (1.4)	2 (0.8)	< 0.001
Cardiac arrest with CPR, *n* (%)	192 (0.6)	138 (1.9)	66 (5.0)	19 (7.8)	< 0.001
Stroke, *n* (%)	41 (0.1)	25 (0.3)	16 (1.2)	5 (2.0)	< 0.001
Pulmonary embolism, *n* (%)	178 (0.6)	72 (1.0)	17 (1.3)	2 (0.8)	< 0.001
ARDS, *n* (%)	142 (0.5)	74 (1.0)	36 (2.7)	8 (3.3)	< 0.001
Pneumonia, *n* (%)	520 (1.7)	243 (3.3)	60 (4.6)	20 (8.2)	< 0.001
Failure-to-rescue, *n* (%)	160 (0.5)	102 (1.4)	49 (3.7)	18 (7.3)	< 0.001

*ARDS* acute respiratory distress syndrome

**Table 4:** IRRs for adverse outcomes in patients with surgically managed isolated traumatic spine injuries

Adverse outcome	RCRI 0	RCRI 1		RCRI 2		RCRI ≥ 3	
		IRR (95% CI)	*P*-Value	IRR (95% CI)	*P*-Value	IRR (95% CI)	*P*-Value
In-hospital mortality	Reference	1.38 (1.18–1.62)	< 0.001	2.25 (1.80–2.80)	< 0.001	3.19 (2.30–4.43)	< 0.001
Cardiopulmonary complication	Reference	1.60 (1.42–1.79)	< 0.001	2.63 (2.22–3.12)	< 0.001	3.27 (2.46–4.34)	< 0.001
Failure-to-rescue	Reference	1.26 (0.96–1.64)	0.090	2.51 (1.77–3.57)	< 0.001	4.27 (2.59–7.02)	< 0.001

IRRs are calculated using Poisson regression models with robust standard errors. Missing values were managed using multiple imputation by chained equations. All analyses were adjusted for age, sex, race, highest abbreviated injury severity score in each region, level of injury, presence and degree of spinal cord injury, level of spine surgery, hypertension, history of peripheral vascular disease, dementia, currently receiving chemotherapy for cancer, disseminated cancer, chronic obstructive pulmonary disease, smoking status, cirrhosis, coagulopathy, drug use disorder, alcohol use disorder, major psychiatric illness, and advanced directives limiting care

*IRR* incident rate ratio; *CI* confidence interval

## Discussion

Given the increasing incidence of traumatic spine injuries among elderly patients [6, 11, 12], who are at a disproportionate risk of death owing to cardiopulmonary causes [4, 5, 13, 14], it is of clinical interest to identify a tool for accurately stratifying patients based on preexisting risk factors. The RCRI, which has previously been validated in a wide range of acute care settings [17–20], could potentially fill this role. In the current investigation, the analyses demonstrate that an elevated cardiac risk, as measured by the RCRI, is associated with a significantly increased risk of in-hospital mortality, cardiopulmonary complications, and FTR in surgically managed traumatic spine injury patients.

These associations are consistent with the results of previous investigations. Several studies that have included individual components of the RCRI have been able to identify them as risk factors for both cardiac and pulmonary complications after spine surgery [26–30]. The same is true for in-hospital mortality following spine surgery, where an association has also been identified with the individual variables included in the RCRI [31–34]. However, while the concept of FTR has developed into a significant quality indicator for postoperative care and has demonstrated high reliability and validity among a range of surgical specialties [35, 36], it has not been as thoroughly studied in traumatic spine injury patients.

Various other tools have been developed that predict the risk of adverse outcomes postoperatively, particularly for the elderly. These tools, which are commonly used in preoperative evaluations, include assessment systems for morbidity and mortality risk such as the American Society of Anesthesiologists physical status classification, the P-POSSUM, SURPAS, the Charlson Comorbidity Index, the machine learning-based POTTER score, and the NSQIP scoring system [37–40]. Nevertheless, a thorough assessment of cardiac risk is necessary to plan additional evaluations and potential risk-mitigating interventions. In situations where the condition is urgent or emergent, patients may not have the chance to undergo such evaluations and may as a substitute require invasive monitoring to identify potential adverse events. The RCRI is widely used for assessing cardiac risk in non-cardiac surgery and can be easily implemented at the bedside or in the emergency room as it only requires six dichotomous variables, without the need for advanced machine learning or artificial intelligence support [15, 16, 21].

Risk stratifying patients can be particularly useful when considering the limited nature of healthcare resources, especially in universal healthcare systems where demand often exceeds supply. Proper allocation of resources, including personnel, operating rooms, equipment, and access to higher levels of care such as intensive care, is always challenging. This is particularly relevant during off-hours when staffing is reduced. The RCRI may in this context be useful for identifying patients who most require critical resources, such as admission to intensive care postoperatively, expedited surgery, or preoperative optimization. Finally, the RCRI could also be considered for determining whether to refrain from surgery in special circumstances.

The RCRI has already been incorporated into multiple guidelines for cardiac protection after surgery [24, 41]. The most recent guidelines from the American College of Cardiology/American Heart Association and the European Society of Cardiology/European Society of Anesthesiology recommend continuing beta-blocker therapy in patients who were already receiving it prior to surgery and also suggest considering initiating beta-blocker therapy in intermediate- to high-risk patients, defined as those with at least two clinical risk factors, an ASA class of at least 3, and at least three factors from the RCRI [24, 41]. Lindenauer et al. found that beta-blockers had a greater protective effect after surgery in patients with a higher RCRI score [42]. This interaction has also been observed in hip fracture patients, who are often frail and suffer from an elevated cardiac risk [19, 20, 43, 44].

The current study has the advantage of using a large national sample population comprised of patients treated at more than 875 trauma centers across the United states [23]. However, it should be noted that it is retrospective in nature and is therefore subject to certain limitations, including the risk of residual confounding, reliance on the accuracy of data recorded in the dataset, as well as the inability to assess variables unavailable in the dataset, such as the cause of death, the use and extent of preoperative optimization, and intraoperative factors.

## Conclusion

The results of the current study suggest that the RCRI may be a useful tool for identifying patients with traumatic spine injuries who are at an increased risk of in-hospital mortality, cardiopulmonary complications, and failure-to-rescue after surgery.
